# Epidemiology of *Taenia saginata* taeniosis/cysticercosis: a systematic review of the distribution in central and western Asia and the Caucasus

**DOI:** 10.1186/s13071-019-3438-3

**Published:** 2019-04-18

**Authors:** Paul R. Torgerson, Aida M. Abdybekova, Gulnara Minbaeva, Zhanna Shapiyeva, Lian F. Thomas, Veronique Dermauw, Brecht Devleesschauwer, Sarah Gabriël, Pierre Dorny, Uffe Christian Braae, Anastasios Saratsis, Lucy J. Robertson, Branko Bobić

**Affiliations:** 1 Section of Epidemiology, Vetsuisse Faculty, University of Zürich, Winterthurerstrasse 270, 8057 Zürich, Switzerland; 2 Kazakh Scientific Veterinary Research Institute, Raiymbek 223, Almaty, Kazakhstan; 3 Government Sanito-Epidemiology Unit, Bishkek, Kyrgyzstan; 4 Scientific-Practical Centre for Sanitary-Epidemiological Expertise and Monitoring, Almaty, Kazakhstan; 5 International Livestock Research Institute, P.O. Box 30709, Nairobi, 00100 Kenya; 6 Institute of Infection and Global Health, University of Liverpool, 8 W Derby St, Liverpool, L7 3EA UK; 7 Department of Biomedical Sciences, Institute of Tropical Medicine, Nationalestraat 155, Antwerp, Belgium; 8 Department of Epidemiology and Public Health, Sciensano, Rue J Wytsman 14, 1050 Brussels, Belgium; 9 Department of Veterinary Public Health and Food Safety, Ghent University, Salisburylaan 133, 9820 Merelbeke, Belgium; 10 Department of Virology, Parasitology and Immunology, Ghent University, Salisburylaan 133, 9820 Merelbeke, Belgium; 11 One Health Center for Zoonoses and Tropical Veterinary Medicine, Ross University School of Veterinary Medicine, P.O. Box 334, Basseterre, Saint Kitts and Nevis; 12 Department of Infectious Disease Epidemiology and Prevention, Statens Serum Institut, 2300 Copenhagen, Denmark; 13 Veterinary Research Institute, Hellenic Agricultural Organisation Demeter, 57001 Thermi, Greece; 14 Parasitology, Department of Food Safety and Infection Biology, Faculty of Veterinary Medicine, Norwegian University of Life Sciences, Adamstuen Campus, Oslo, Norway; 15 Centre of Excellence for Food and Vector-borne Zoonoses, Institute for Medical Research, University of Belgrade, Dr Subotića 4, Belgrade, 11000 Serbia

**Keywords:** *Taenia saginata*, Cestode, Beef tapeworm, Bovine cysticercosis, Taeniosis, Central Asia, Iran, Turkey, Caucasus

## Abstract

**Background:**

The zoonotic parasite *Taenia saginata* transmits between humans, the definitive host (causing taeniosis), and bovines as the intermediate host (causing cysticercosis). Central and western Asia and the Caucasus have large cattle populations and beef consumption is widespread. However, an overview of the extent of human *T. saginata* infection and bovine cysticercosis is lacking. This review aims to summarize the distribution of *T. saginata* in this region.

**Methods:**

A systematic review was conducted, that gathered published and grey literature, and official data concerning *T. saginata* taeniosis and bovine cysticercosis in central and western Asia and the Caucasus published between January 1st, 1990 and December 31st, 2018. Where no data were available for a country within this period, published data from 1985–1990 were also accessed.

**Results:**

From 10,786 articles initially scanned, we retrieved 98 full-text articles from which data were extracted. In addition, two unpublished datasets were provided on the incidence of human taeniosis. Data for human taeniosis and bovine cysticercosis were found for all countries except Turkmenistan. Human taeniosis prevalence varied from undetected to over 5.3%, with regional variations. Where bovine cysticercosis was detected, prevalences varied from case reports to 25%.

**Conclusions:**

The public health burden of *T. saginata* is assumed to be small as the parasite is of low pathogenicity to humans. However, this review indicates that infection continues to be widespread and this may result in a large economic burden, due to the resources utilized in meat inspection and condemnation or processing with subsequent downgrading of infected carcasses.

**Electronic supplementary material:**

The online version of this article (10.1186/s13071-019-3438-3) contains supplementary material, which is available to authorized users.

## Background

The area of central and western Asia and the Caucasus includes countries of the former Soviet Union (Armenia, Azerbaijan, Georgia, Kazakhstan, Kyrgyzstan, Tajikistan, Turkmenistan and Uzbekistan) in addition to Iran and Turkey. Diseases caused by cestode zoonoses such as *Echinococcus* spp. have been known to emerge or re-emerge in this region [1, 2]. This emergence may be explained, at least partially, by socio-economic changes resulting from the collapse of the former Soviet Union [3], which resulted in privatization of large collective farms, closure of meat-processing plants, and deterioration in veterinary public health services.

*Taenia saginata*, the beef tapeworm, is an important cyclo-zoonotic cestode, with a worldwide distribution. The adult tapeworm develops in the human intestine, producing eggs that are either excreted directly in the faeces or in intact egg-containing proglottids [4]. Cattle, the usual intermediate hosts of the parasite, acquire the infection by the ingestion of eggs, and subsequent migration of the oncosphere *via* the bloodstream to striated muscles results in the development of a protoscolex containing the cysticercus, the metacestode stage. The success and widespread distribution of this parasite can be associated with a range of factors related to the definitive and intermediate hosts as well as to the outer environment in order for its life-cycle to be maintained. This includes both dietary habits (consumption of raw or undercooked cysticerci-infected meat) and sanitary education level of farm workers, as well as appropriate treatment and disposal of sewage [5].

The clinical effects of *T. saginata* on humans are relatively trivial; usually at most limited to mild gastrointestinal signs and anal pruritus. With almost no fatalities and a very low disability weight, the global burden of disease due to *T. saginata* is vanishingly low, despite it being a common parasitic infection in some low-income countries. There are, however, occasional case reports of gastrointestinal pain and discomfort or appendicular taeniosis. These include case reports from Iran, which is within the region of the present study and have been documented by Moazeni et al. [6]. The direct economic costs in terms of human disease are consequently also very low and limited to the cost of diagnosis treatment [7]. In cattle, there are limited studies on the economic costs of infection on production losses. However, in most high-income countries, inspection of beef for the presence of bovine cysticercosis is compulsory. Carcasses shown to be infected may be condemned or downgraded and refrigerated [8]. It is this downgrading that can cause substantial economic losses and infection of cattle with *T. saginata* may have adverse effects on trade [9].

There are over 48 million head of cattle in central and western Asia and the Caucasus [10], hence the aim of the present study is to provide a systematic review of the prevalence of *T. saginata* taeniosis and bovine cysticercosis, for the use of researchers and policy makers alike.

## Methods

### Search strategy

This systematic review was compiled based on the PRISMA guidelines [11] (Additional file 1: Table S1). The study focused on the region of central and western Asia and the Caucasus. It did not include countries traditionally regarded as Middle East countries or the Russian Federation as these countries are covered in accompanying articles [12, 13]. The study area did include the following countries: Armenia, Azerbaijan, Georgia, Iran, Kazakhstan, Kyrgyzstan, Turkey, Turkmenistan, Tajikistan and Uzbekistan. Databases were searched for information relating to the occurrence, prevalence, and geographical distribution of human taeniosis due to *T. saginata* and bovine cysticercosis for the period between the 1st of January 1990 and 31st of December 2018.

A specific combination of search words was used to search both for published papers and grey literature (MSc/PhD theses, reports etc.) in three international bibliographic databases (PubMed and Web of Science, Google Scholar). The search terms were as follows: (cysticerc* OR cisticerc* OR “C. bovis” OR taenia* OR tenia* OR saginata OR taeniosis OR teniosis OR taeniasis OR ténia OR taeniid OR cysticerque OR Taeniarhynchus) AND (above-mentioned countries separated by the operator “OR”). In addition, WHO IRIS (http://apps.who.int/iris/) were searched by using a combination of three search words (i.e. *Taenia* and *saginata* or cysticerc*). As several of the countries were from the former Soviet Union, we also searched Russian databases cyberleninka (https://cyberleninka.ru/) and elibrary.ru (https://elibrary.ru).

We used English language search terms, but for Google Scholar (GS) we also used the search terms in Russian, Turkish and Persian. For cyberleninka and elibrary.ru we used search terms in Russian. GS searches in English usually identified very large numbers of articles. For example, “Intestinal Parasites Turkey” revealed 2400 results compared to 1200 in Web of Science. Thus for GS searches we searched by relevance and examined the first 300 returned search items [14].

Reference lists of reviews on the topic and of selected papers were screened and additional relevant records were added to the database. Some unpublished data were also provided from central Asia in the context of notification to the epidemiological services and unpublished reports from institutes.

### Selection criteria

Upon compilation of search results from the different databases, duplicate records were removed. Titles and abstracts were then screened for relevance, applying the following exclusion criteria: (i) studies concerning a parasite other than *T. saginata*; (ii) studies conducted outside the study area; (iii) studies published outside the study period, unless no other data were available for that country; (iv) studies reporting results outside the scope of the review question (e.g. review, experiment, intervention trial); and (v) duplicated data. After the screening process, full text articles were evaluated, and data extracted. The list of articles from which data were extracted is given in Additional file 2: Text S1. Exceptionally, some data from the late 1980s were included for bovine cysticercosis from Kazakhstan and Azerbaijan as no other data were found.

### Data extraction and generation

Data from included records were extracted. In reports where the numerator and denominator of the study sample were available, prevalence data were calculated, if not already provided. Data at the country level or district level were then mapped with prevalence estimates of human taeniosis and bovine cysticercosis.

## Results

### Search results

**Fig. 1:**
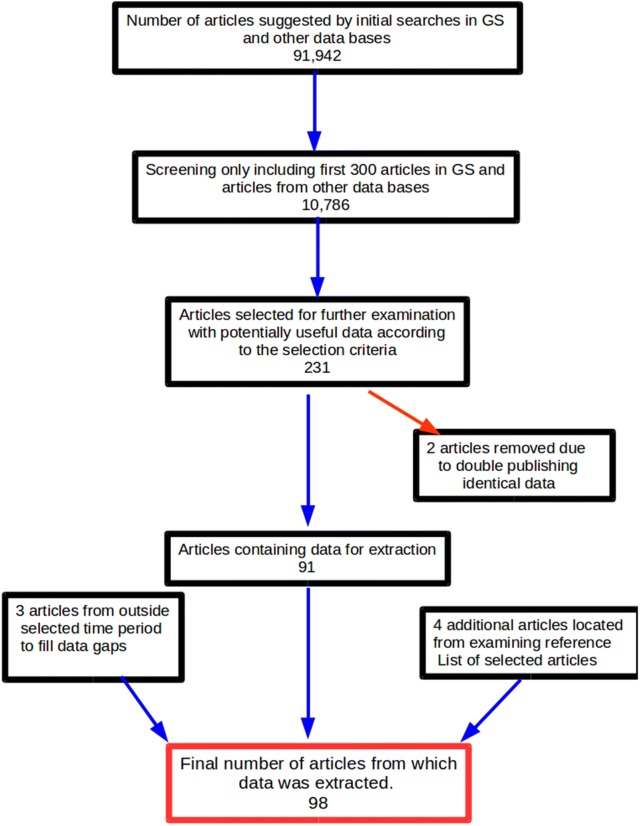
Flow chart indicating process for selection of reports with usable data

**Fig. 2:**
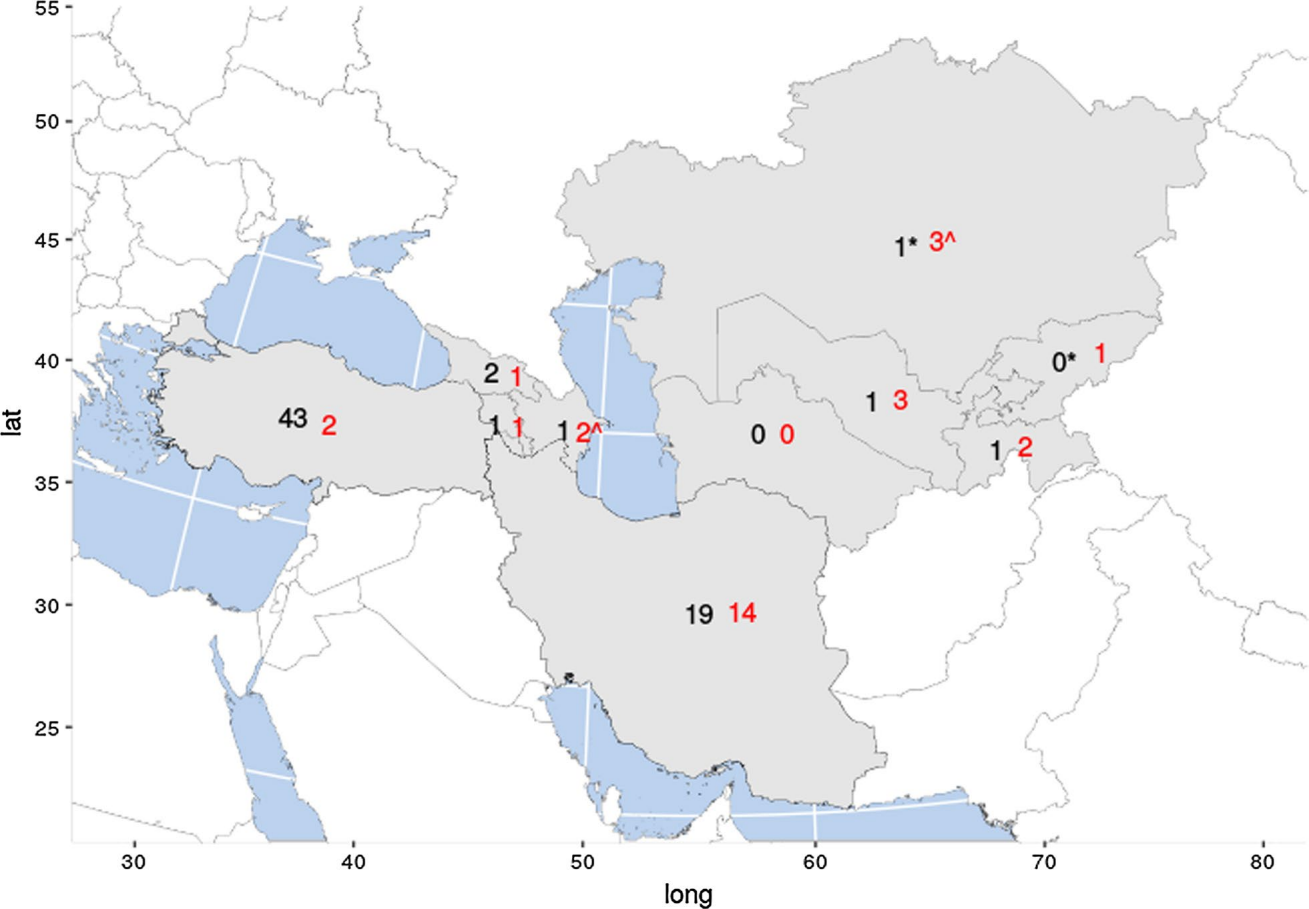
Numbers of reports (black human taeniosis, red bovine cysticercosis) from each country from which data were extracted. *Unreported official data used. ^Data from 1985–1989 used

A total of 91,948 records were suggested from the database search. However as only the first 300 GS articles in each search were examined [14], this was reduced to 10,786. After further screening of the titles, 231 articles were further examined. Of these, 96 full-text articles contained data that were eligible for inclusion in the study. However, 2 of these articles duplicated the same data but were published in 2 articles (double publishing), so only one of each of these articles were included. A further 4 articles were found by consulting the reference list of selected articles. This resulted in 98 articles from which data were extracted (Fig. 1). The list of full-text articles or other data sources from each country is given in Additional file 2: Text S1. This includes 3 reports published between 1985 and 1989 that filled data gaps in more recent literature. The number of articles and data sources located for each country are illustrated in Fig. 2.

### Geographical distribution

**Fig. 3:**
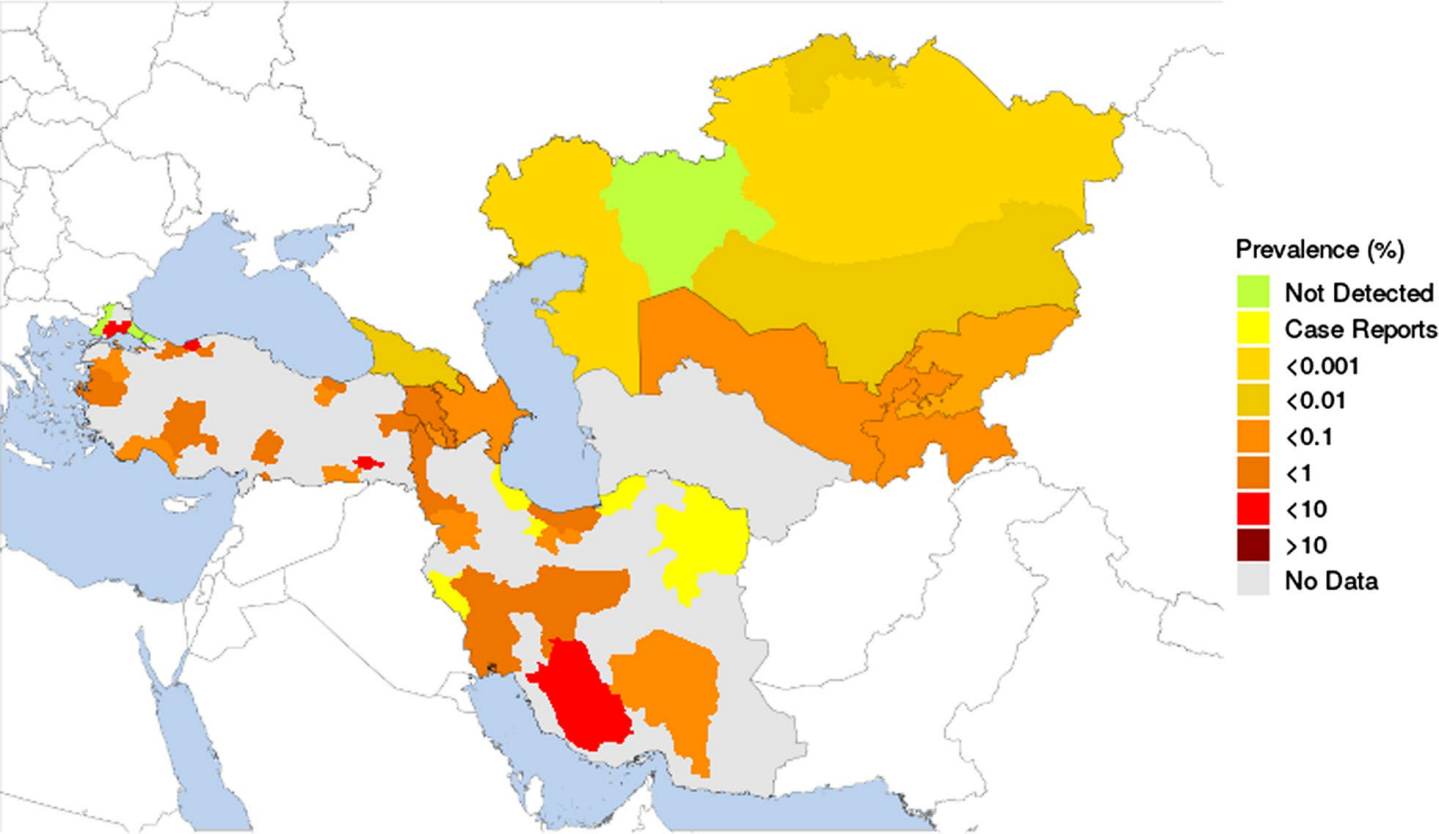
Estimated prevalence of human taeniosis based on extracted data

**Fig. 4:**
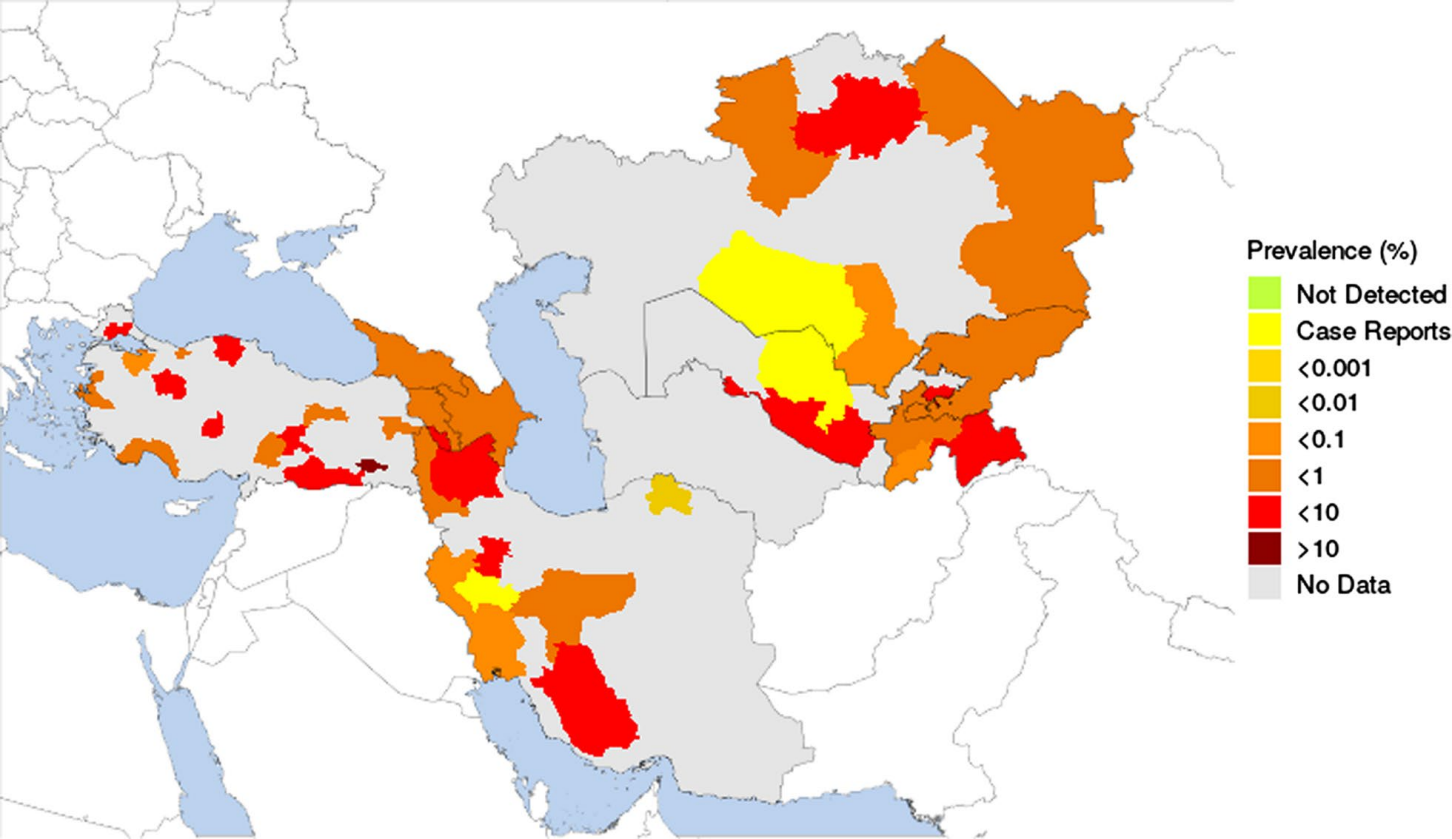
Estimated prevalence of bovine cysticercosis based on extracted data

Data were obtained for all countries except Turkmenistan. It appears that *T. saginata* is endemic throughout the study region. Figure 3 illustrates the reported prevalences of human taeniosis calculated from data provided in the selected articles and other sources. The highest prevalences of human taeniosis appear to occur in parts of Turkey (up to 5.3%) and this is mirrored by relatively high prevalences of bovine cysticercosis (up to 25%) (Fig. 4).

## Discussion

Our systematic review indicates that *T. saginata* is endemic throughout the study area. Only in Turkmenistan were we unable to find any data. Nevertheless, the parasite is almost certainly present in this country as beef consumption is widespread and the parasite is endemic in all neighbouring countries.

In several countries of the former Soviet Union, human *T. saginata* infection is notifiable. For Kazakhstan and Kyrgyzstan, the country and regional estimates of human taeniosis due to *T. saginata* were based on these data. Taeniosis due to *T. solium* is also notifiable, but there were very few cases reported in the databases. Taeniosis due to *T. saginata* was reported as taeniarhynchiasis, whilst that for *T. solium* as taeniosis. This reflects the fact that Russian parasitologists still classify *T. saginata* and *T. solium* as being from different genera (*Taeniarhynchus* and *Taenia,* respectively). However, the data do not indicate the method used to differentiate between the two taeniid infections. Except for Georgia, Armenia, and the Russian minority population in Kazakhstan, most of the human population of the region covered by this report are Muslims and hence pork consumption is very low or negligible, which makes transmission of *T. solium* unlikely. The same argument can be used for *T. asiatica*, which is primarily transmitted to humans from pork. Most of the published reports of taeniosis and bovine cysticercosis came from two countries: Iran and Turkey.

Diagnosis of intestinal parasites typically relies on the microscope detection of transmission stages in human faecal samples. It was more often the case that taeniid eggs were reported rather than a specific diagnosis of *T. saginata* or *T. solium* taeniosis. However, because most of these reports were from Iran or Turkey, where pork consumption is very low, it can be assumed that these were eggs of *T. saginata*. Nevertheless, confirmation would either need PCR analysis of the eggs or morphological analysis of proglottids that might also be present in the faeces. The prevalence is also likely to be underestimated because of the poor sensitivity of microscopy and the asymptomatic nature of infection [15].

The most data-rich country, Iran, actually has the lowest per capita consumption of beef, at 3.6 kg per annum. Turkey has intermediate levels of beef consumption, whilst the highest levels of beef consumption are in a number of the newly-independent states of the former Soviet Union [10] (see Table 1). Furthermore, poor sanitation would also be expected to be associated with the transmission of *T. saginata.* Of this group of countries, Turkmenistan has the highest indicator of mortality associated with poor sanitation with a mortality of 4 per 100,000 per year [16] (Table 1). Therefore, although data for taeniosis/cysticercosis were not available for Turkmenistan, the high beef consumption combined with poor sanitation indicators suggests that *T. saginata* taeniosis and bovine cysticercosis are both likely to be present. Factors other than poor sanitation, such as the use of sewage sludge on grazing pasture or access to surface water contaminated with effluent, are also important in transmission, as has been reported in European counties such as Switzerland, Belgium and Denmark [17–19].

**Table 1:** Annual per capita consumption of beef and mortality associated with poor sanitation. Data from [10, 16]

Country	Annual per capita consumption of beef (kg)	Mortality associated with poor sanitation (annual per 100,000)
Armenia	19.7	0.2
Azerbaijan	12.7	1.1
Georgia	6	0.2
Iran	3.6	1
Kazakhstan	23.4	0.4
Kyrgyzstan	14.9	0.8
Tajikistan	4.4	2.7
Turkey	11.6	0.3
Turkmenistan	26.1	4
Uzbekistan	28.2	0.4

We generally found prevalences in both cattle and humans to be higher than that reported in western Europe [20] comparable with eastern Europe [21], South America [22] and the Middle East [12] but lower than in Africa [23]. This may be related to standards of sanitation and veterinary supervision of the slaughter of livestock. Both of these are likely to be of a lower standard in lower income countries such as in Africa.

For this systematic review, we relied heavily, although not entirely, on Google Scholar (GS). There is some debate as to if GS has sufficient coverage as the sole search engine; see Giustinin & Boulos [24] and Gehanno et al. [25] for conflicting views on the use of GS. However, GS does appear to be efficient at finding much, but not all, grey literature [14]. In this review, the other search engines failed to find additional material that was not found by GS. In addition, terms in scripts other than Latin can be used as search terms. Putting search terms into Russian (Cyrillic) or Persian (modified Arabic script) resulted in retrieving of eight additional articles (six in Russian and two in Persian) with usable data that were not found by any other method (see Additional file 2: Text S1). One disadvantage of GS is that the number of hits returned can be vast, and hence it is recommended to make the search by relevance and only examine the first 300 records returned [14]. This was particularly marked when using the non-specific search term “intestinal parasites”. This resulted in retrieving a number of papers reporting *Taenia saginata* prevalences in humans that were not found with other search terms. However, it also returned large amounts of literature on intestinal parasites of domestic animals and even wildlife which were not relevant to the present review.

## Conclusions

The present study provides an up-to-date overview of the distribution of this parasite across central and western Asia and the Caucasus in spite the limitations outlined. The high prevalence of taeniosis and bovine cysticercosis in Turkey suggests that substantial economic burden may be experienced due to this parasite and that consideration should be given to reducing exposure of cattle to human faecal material within this country.

## Additional files

**Additional file 1: Table S1.** PRISMA checklist.

**Additional file 2: Text S1.** References and other sources from which data were extracted.

## Abbreviations

GS: Google Scholar
PRISMA: Preferred Reporting Items for Systematic Reviews and Meta-Analyses
WHO IRIS: World Health Organization International Repository for Information Sharing
