# Supplementary material for: Epidemiology of Taenia saginata taeniosis/cysticercosis: a systematic review of the distribution in central and western Asia and the Caucasus
Source: Parasit Vectors. 2019 Apr 18;12:175. doi: 10.1186/s13071-019-3438-3 (PMC6472068; doi:10.1186/s13071-019-3438-3)
Supplement: Supplementary file 2 — Additional file 2: Text S1. References and other sources from which data were extracted. [file 13071_2019_3438_MOESM2_ESM.docx]

**Additional file 2: Articles from which data were extracted**

For articles found only with non-English search terms (Google Scholar, Cyberleninka, elibrary.ru), the titles are given in the original language followed by an English translation

**Human Infection**

Armenia (1 report)

Avetisian, L. M. (2004). Epidemiological surveillance of parasitic diseases in the republic of Armenia. Meditsinskaia Parazitologiia I Parazitarnye Bolezni, 1, 21-24.

Azerbaijan (1 report)

Nadzhafov I. G. and Darchenkova, N. N.(1990) An attempt at dividing the territory of Azerbaijan according to for infestation with Taeniorhynchus. Meditsinkkaya Parasitolgiya I Parazitarnaye Bolezni, 2, 20-24

Georgia (2 reports)

Khalafli Kh. (2009) epidemiolgy aspects intestinal parasitosis among the population of Baku. Georgian Medical News, 3, 84-87

Зиракишвили, Л. М., Иашвили, Н. Д., Иосава, М. Д., Поцхверия, Ш. О., & Месхи, М. В. (2009). Эпидемиологическая и эпизоотологическая характеристика трихинеллеза и тениаринхоза в Грузии. Теория и практика паразитарных болезней животных, 10, 172-176

[Zirakishvily L.M., Iashvily N.D., Iosava M.D., Pochveria Sh.O., Meschy M.V. Georgian S.S. (2009). Epidemiological and epizootological characteristics of Trichinella spp. and Taeniarhynchus saginatus infections in Georgia.Theory and Practice of Parasitic Diseases of Animals, 10, 172-176]

Iran (19 reports)

Akhlaghi, L., Shamseddin, J., Meamar, A. R., Razmjou, E., Oormazdi, H. (2009) Frequency of intestinal Parasites in Tehran. Iranian Journal of Parasitology, 4, 44-47.

Fares Bahrami, Ali Haghighi, Ghasem Zamini, Mohammad Bagher Khadem-Erfan and Eznolla Azargashb. Prevalence and associated risk factors of intestinal parasitic infections in Kurdistan province, northwest Iran. Cogent Medicine (2018), 5, 150

Hafezi Ahmadi, M., & Seifmanesh, H. (2011). Taeniasis caused appendicitis without local tenderness: A rare case. *Hospital Chronicles*, *6*(4), 207-209.

Heidari, Aliehsan, Enayatollah Kalantar, Parviz Fallah, Mohammad Hossein Dehghan, Sadegh Saedi, Moniereh Sezavar, and Morteza Ghoghaee. "Case Record of a Teaching Hospital in Karaj; A 35-Year Old Man With Taenia saginata Infection Treated With Niclosamide." *International Journal of Enteric Pathogens* 4, no. 4 (2016): 9-37109.

Kia , E. B.,Masoud, J., Yalda, A., Mahmoudi, M., Farahani, H. (2005) Study on Human Taeniasis by Administering Anti-Taenia Drug. Iranian J Publ Health, 2005, Vol. 34, No. 4, pp.47-50

Kheirandish, F., Tarahi, M. J.,Haghighi, A.,Nazemalhosseini- Mojarad, E., Kheirandish, M. (2011) Prevalence of Intestinal Parasites in Bakery Workers in Khorramabad, Lorestan Iran. Iranian J Parasitol, 6, 76-83

Jasem Saki, Shahram Khademvatan, Masoud Foroutan-Rad, and Marziyeh Gharibzadeh. 2017. Prevalence of Intestinal Parasitic Infections in Haftkel County, Southwest of Iran. Int J Infect. 2017 October; 4(4):e15593.

Rasool Jafari, Forough Sharifi, Bahram Bagherpour, Marzieh Safari. 2016. Prevalence of intestinal parasites in Isfahan city, central Iran, 2014. Journal of Parasitic Diseases, 40, 679-682.

Maryam Barkhori Mahni, Mostafa Rezaeian, Eshrat Beigom Kia, Ahmad Raeisi, Khadijeh Khanaliha, Fatemeh Tarighi, Bahare Kamranrashani (2016). Prevalence of Intestinal Parasitic Infections in Jiroft, Kerman Province, Iran. Iran J Parasitol: Vol. 11, No. 2, Apr -Jun 2016, pp. 232-238

Nasiri, V., Esmailnia, K., Karim, G., Nasir, M. and Akhavan, O., 2009. Intestinal parasitic infections among inhabitants of Karaj City, Tehran province, Iran in 2006-2008. The Korean Journal of Parasitology, 47(3), p.265.

Neghab, M., Moosavi, S., & Moemenbellah-Fard, M. D. (2006). Prevalence of intestinal parasitic infections among catering staff of students canteens at Shiraz, southern Iran. *Pak J Biol Sci*, *9*(14), 2699-703.

Pagheh, A.S., Sharif, M., Daryani, A., Yazdani-Charati, J., Nazar, E., Asfaram, S., Hosseini, S.A., Tork, M., Soosaraie, M., Syadatpanah, A. and Gholami, S., 2018. A cross-sectional analysis of intestinal parasitic infections among the general population in north of Iran. The Journal of Infection in Developing Countries, 12(02), pp.120-126

Sayyari, S., Imanzadeh, F., Bagheri, S. A., Karami, H., Yaghoobi, M. 2005. Prevalence of intestinal parasitic infections in the Islamic Republic of Iran. Eastern Mediterranean Health Journal, 11, 377-383

Soosaraei, M., Alizadeh, S., Fakhar, M., Banimostafavi, E. S., & Hezarjaribi, H. Z. (2017). Intestinal perforation and peritonitis due to Taenia saginata: A case report from Iran. Annals of medicine and surgery, 24, 74-76.

Shafaghi, A., Rezayat, K. A., Mansour-Ghanaei, F., & Maafi, A. A. (2015). Taenia: an uninvited guest. The American journal of case reports, 16, 501.

Sheikhian, M. R. (2013). A common worm in a rare place. *Iranian journal of public health*, *42*(11), 1321.

Tork, M., Sharif, M., Charati, JY, Nazar, I., Hosseini, SA. (2016)Prevalence of Intestinal Parasitic Infections and Associated Risk Factors In West of Mazandaran Province, Iran. J Mazandaran Univ Med Sci 2016; 26(137): 81-88 (Persian).

حضرتی تپه, مستقیم, مهیار, عباسی, فریدونی, جاوید, & حسن‌زاده. (2004). بررسی فراوانی آلودگی‌های انگلی روده‌ای در مراجعه کنندگان به آزمایشگاه کلینیک ویژه دانشگاه علوم پزشکی ارومیه طی سال‌های 81-87. مجله دانشکده

پرستاری و مامایی ارومیه, 2(1), 0-0.‎

[Hazrati, Mahyar, Abbasi, Fereiduni, Javid, & Hassanzadeh. (2004). Frequency of intestinal parasitic contamination in the referrals to Urmia University of medical sciences clinical laboratory during the years of 81-87. Journal of Urmia Nursing and Midwifery Faculty, 2 (1), 0-0.]

مذهب جعفري, کميل, منيعي, مراغي, & شريف. (2012). بررسي گذشته¬ نگر فراواني آلودگي با انگل‌هاي روده‌اي در بيماران مراجعه¬ كننده به آزمايشگاه مرکزي بيمارستان بزرگ دزفول در سال 1389-1390.

[Mohammad Maniey 1 , Sharif Maraghi 2 , Komeil Mazhabjafari 3* (2012). A retrospective study of the frequency of intestinal parasites infection in patients referring to the central laboratory of Dezful Hospital in 2011-2011. Jundishapur Journal of Health Sciences, 4 (2), 31-38.]

Kazakhstan (1 report and additional unpublished data)

Шайзадина, Ф. М., Брицкая, П. М., Култанов, Б. Ж., Кантимиров, М. Р., Алышева, Н. О., & Беменде, Б. (2013). Эпидемиологическая ситуация по гельминтозным инвазиям среди населения в центральном Казахстане. Международный журнал прикладных и фундаментальных исследований, (5), 147-148.

[Shayzadina, F. M., Britskaya, P. M., Kultanov, B. Zh., Kantimirov, M. R., Alysheva, N. O., & Bemende, B. (2013). Epidemiological situation of helminthic invasions among the population in central Kazakhstan. International Journal of Applied and Fundamental Research, (5), 147-148.]

Unpublished Data supplied by the Government Sanito-Epidemiological Services

Kyrgyzstan

Unpublished Data supplied by the Government Sanito-Epidemiological Services

Tadjikistan (1 report)

Murtazoev D.M., Valiev H.G., Pulotov M.B., Faizullaev U.F. (2015) Epidemiological situation and problems in control of Taeniarhynchus saginata infection. Theory and Practice of Parasitic Diseases of Animals,16, 276-278

Turkmenistan

No articles found

Turkey (43 reports)

Akdemir, C. and Helvaci, R. (2007) Evaluation of Parasitological Laboratory Results of a Group of People Older Than 15 Years of Age in Kutahya. Türkiye Parazitoloji Dergisi, 31 (1): 37-40

Alver, O., Özakin, C., & Okan, T. Ö. R. E. (2012). The distribution of intestinal parasites detected in the Uludag University Medical Faculty Hospital between 2009-2010. Türkiye Parazitolojii Dergisi, 36(1), 17.

Alver, O., & Töre, O. (2006). Uludağ Üniversitesi Tıp Fakültesindeki bağırsak parazit olgularının prevalansı ve dağılımı. Türkiye Parazitol Derg, 30(4), 296-301.

Alver, O., Oral, B., & Okan, T. Ö. R. E. (2011). The distribution of intestinal parasites detected in the Uludag University Medical School Hospital between 2005 and 2008. Türkiye Parazitolojii Dergisi, 35(4), 194

Alver, O., Heper, Y., Ercan, I., Akalin, H., Tore, O. (2011). Prevalence of ıntestinal parasites in Bursa Province of Turkey and assessment of enzyme-linked immunosorbent assays (ELISA) and three microscopic methods in the diagnosis of Entamoeba histolytica/Entamoeba dispar. African Journal of Microbiology Research Vol. 5(12), pp. 1443-1449

Atas, A. D., Alim, A., Atas, M. (2008) Distribution of Intestinal Parasites in Patients Presenting at the Environmental-Food and Medicine Analysis Laboratory of Sivas Municipality during the Years 1993-2006. Türkiye Parazitoloji Dergisi, 32 (1): 59-64,

Atas, A. D., and Kuscuoglu, S. (2010) Distribution of Intestinal Parasites Detected in the Tokat Public Health Laboratory during the Period from January 2007- December 2009. Türkiye Parazitoloji Dergisi, 34 (3): 161-166

Ataş, A. D., Alim, A., Ataş, M., & Artan, M. O. (2008). The investigation of intestinal parasites in two primary schools in different social-economic districts of the city of Yozgat, Turkey. Turkiye parazitolojii dergisi, 32(3), 261-265.

Celiksov, A., Acioz, M., Degerli, S., Alim, A., Aygan, C. (2005) Egg positive rate of Enterobius vermicularis and Taenia spp. by cellophane tape method in primary school children in Sivas, Turkey. The Korean Journal of Parasitology Vol. 43, No. 2. 61-64

Celiksoz, A., Guler, N., Guler, G., Yasemin Oztor, A., Degerli, S. (2005). Prevalence of Intestinal Parasites in Three Socioeconomically-different Regions of Sivas, Turkey. Journal of Health, Population and Nutrition, Vol. 23, No. 2 (June 2005), pp. 184-191

Ciftci, I. H., Cetinkaya, Z., Demirol, T., Kiyildi, N., Demirturk, N., & Altindis, M. (2004). Distribution of intestinal parasitosis in the Mimar Sinan and Atatürk primary schools in Bayat, Afyon, Turkey. Turkiye Parazitol Derg, 28(4), 215-217.

Culha, G., Sangun, O., Incecik, F. (2005) Distribution of Intestinal Parasites in Children Aged between 0 and 14 Presenting at the Laboratory of Parasitology of the Mustafa Kemal University Medical School. Türkiye Parazitoloji Dergisi, 29 (4): 255-257

Culha, G. (2006) The Distribution of Patients with Intestinal Parasites Presenting at the Parasitology Laboratory of the Mustafa Kemal University Medical Faculty. Türkiye Parazitoloji Dergisi, 30 (4): 302-304

Dahin, I., Yazar, S., Yaman, O., Gozkenc, N. (2006) Investigation of Intestinal Parasites in Residents of the Kayseri Karpuzsekisi Basin. Türkiye Parazitoloji Dergisi, 30 (3): 178-180

Dagci, H., Kurt, Ö., Demirel, M., Östan, I., Azizi, N.R., Mandiracioglu, A., Yurdagül, C., Tanyüksel, M., Eroglu, E. and Ak, M., 2008. The prevalence of intestinal parasites in the province of Izmir, Turkey. Parasitology research, 103(4), p.839.

Dergerli, S., Ozcelik, S., Celiksoz, A. (2005)The Distribution of Intestinal Parasites in Patients Presenting at the Parasitology Laboratory of the Cumhuriyet University. Türkiye Parazitoloji Dergisi, 29 (2): 116-119

Değerlı, S., Ozçelık, S., & Celıksöz, A. (2005). The distribution of intestinal parasites in patients presenting at the Parasitology Laboratory of the Cumhuriyet University. Turkiye parazitolojii dergisi, 29(2), 116-119.

Değirmenci, A., Sevil, N., Güneş, K., Yolasiğmaz, A., & Turgay, N. (2007). Distribution of intestinal parasites detected in the parasitology laboratory of the Ege University Medical School Hospital, in 2005. Turkiye parazitolojii dergisi, 31(2), 133-135.

Doğan, N., Demirüstü, C., & Aybey, A. (2008). The prevalence of intestinal parasites according to the distribution of the patients' gender and parasite species for five years at the Osmangazi University Medical Faculty. Turkiye parazitolojii dergisi, 32(2), 120-125.

Gulmez, D., Saribas, Z., Akyon, Y., Erguven, S. (2013) The results of Hacettepe Univerwsity Faculty of medicine Parasitology Laboratory in 2003-2012: Evaluation of 10 years. Turkiye Parazitol Derg 37: 97-101

Gundez, T., Demirel, M. M., Inceboz, T., Tosun, S., Yereli, K. (2005). Prevalence of Intestinal Parasitosis in Children with Gastrointestinal Symptoms Associated with Socio-Economic Conditions in Manisa Region. Türkiye Parazitoloji Dergisi, 29 (4): 264-267

Kaya, S., Demirci, M., Demirel, R., Cicioglu, B., Ozturk, M., & Sirin, C. (2004). Prevalence of intestinal parasites in the city of Ispar‐ta. Turkiye Parazitol Derg, 28(2), 103-105.

Kırkoyun, H. U., Akgül, O., Purisa, S., & Oner, Y. A. (2014). Twenty-five years of intestinal parasite prevalence in İstanbul University, İstanbul Faculty of Medicine: a retrospective study. Turkiye parazitolojii dergisi, 38(2), 97-101.

Koroglu, M., Yakupogullari, Y., Turhan, R. (2007) A Retrospective Analysis of the Results of a Seven-Year Parasitological Examination of Stools from Malatya State Hospital. Türkiye Parazitoloji Dergisi, 31 (3): 201-204

Koksal, F., Baslanti, I., Samasti, M. (2010) A Retrospective Evaluation of the Prevalence of Intestinal Parasites in Istanbul, Turkey. Türkiye Parazitoloji Dergisi, 34 (3): 166-171

Kurtoglu, M. G., Korkoca, H., Cicek, M., Tas Cengiz, Z. (2007). Prevalence of Intestinal Parasites Among Workers in Food Sector in Van Regıon. Türkiye Parazitoloji Dergisi, 31 (4): 309-312

Malatyali, E., Ozcelik, S., Celiksöz, A., Değerli, S., & Yildirim, D. (2008). The frequency of intestinal parasites in primary school children in urban and rural regions. Turkiye parazitolojii dergisi, 32(1), 54-58

Okyay, P., Ertug, S., Gultekin, B., Onen, O. and Beser, E., 2004. Intestinal parasites prevalence and related factors in school children, a western city sample-Turkey. BMC public health, 4(1), p.64.

Ozgümüş, O. B., & Efe, U. (2007). Distribution of intestinal parasites detected in Camlihemşin healthcare center during the period from July 2005 to January 2007. Turkiye parazitolojii dergisi, 31(2), 142-144.

Ozgümüş, O. B., & Karaoğlu, S. A. (2007). Screening of intestinal parasites of children in special day nurseries in the city of Rize. Turkiye parazitolojii dergisi, 31(3), 205-207.

Ozyurt, M., Kurt, O., Yaman, O., Ardiç, N., & Haznedaroğlu, T. (2007). Evaluation of intestinal parasites in a period of four years in the coprology laboratory of a training hospital. Turkiye parazitolojii dergisi, 31(4), 306-308.

Sahin, I., Yazar, S., Yaman, O., & Gözkenç, N. (2006). Investigation of intestinal parasites in residents of the Kayseri Karpuzsekisi Basin. Turkiye parazitolojii dergisi, 30(3), 178-180.

Simsek, Z., Koruk, I., Copur, A. C., & Gürses, G. (2009). Prevalence of Staphylococcus aureus and intestinal parasites among food handlers in Sanliurfa, Southeastern Anatolia. Journal of Public Health Management and Practice, 15(6), 518-523.

Selek, M. B., Bektöre, B., Karagöz, E., Baylan, O., & Özyurt, M. (2016). Distribution of Parasites Detected in Stool Samples of Patients Admitted to Our Parasitology Laboratory during a Three-Year Period between 2012 and 2014. Turkiye parazitolojii dergisi, 40(3), 137-140.

Tamer, G. S., Calişkan, S., & Willke, A. (2008). Distribution of intestinal parasites among patients who presented at the parasitology laboratory of the Kocaeli University School of Medicine Hospital. Turkiye parazitolojii dergisi, 32(2), 126-129.

Turhan, E., Inandi, T., Cetin, M., & Taş, S. (2009). The distribution of intestinal parasites in children living in orphanages in Hatay, Turkey. Turkiye parazitolojii dergisi, 33(1), 59-62.

Ulukanligil, M., Seyrek, A. (2003) Demographic and parasitic infection status of schoolchildren and sanitary conditions of schools in Sanliurfa, Turkey. BMC Public Health, 3:29

Usluca, S., Yalçin, G., Over, L., Tuncay, S., Sahin, S., Inceboz, T., & Aksoy, U. (2006). The distribution of intestinal parasites detected in the Dokuz Eylul University Medical Faculty Hospital between 2003 and 2004. Turkiye parazitolojii dergisi, 30(4), 308-312.

Uzun, A. (2004). The investigation of intestinal parasites in five primary schools in different areas of the city of Diyarbakir, Turkey. Acta Parasitol Turc, 28(3), 133-135.

Yaman, O., Yazar, S., Ozcan, H., Cetinkaya, U., Gözkenç, N., Ateş, S., & Sahin, I. (2008). Distribution of intestinal parasites in patients presenting at the parasitology laboratory of the medical school of Erciyes University between the years of 2005 and 2008. Turkiye parazitolojii dergisi, 32(3), 266-270.

Yazar, S., Yaman, O., Gozkens, N., Saghin, I. (2005) Distribution of Intestinal Parasites among Patients Who Presented at the Department of Parasitology of the Erciyes University Medical School. Türkiye Parazitoloji Dergisi, 29 (4): 261-263,

Yapici, F., Sönmez, G. T., & Arisoy, E. S. (2008). The distribution of intestinal parasites and their causative factors in children. Turkiye parazitolojii dergisi, 32(4), 346-350.

Yilmaz, H., Akman, N., & Göz, Y. (1999). Distribution of intestinal parasites in two societies with different socio-economic status in Van. Eastern Journal of Medicine, 4(1), 16-19.

Uzbekistan (1 report)

Dzhumaev, M. D. (1996) The organization of the control of taeniarhynchiasis in Bukhara Provionce. Meditsinkaia Parasitolgiia I Parazitarnye Bolezni, 3, 55-56. [Russian]

**Cattle Infection**

Armenia (1 report)

Манучарян, Елена Георгиевна, 1993. Прогнозирование эпизоотической и эпидемической ситуаций по гельминтозоонозам в Армении. кандидата ветеринарных наук. Москва, 1993. Диссертации о Земле http://earthpapers.net/prognozirovanie-epizooticheskoy-i-epidemicheskoy-situatsiy-po-gelmintozoonozam-v-armenii#ixzz5eZ43ZlHE

[Manuchrya, E. G. (1993) Predictions for epizootiological and epidemiological situation for helminth zoonoses in Armenia. Dissertation Candidate Veterinary Sciences, Moscow.]

Azerbaijan (2 reports)

Фархадов, к. Т. О. (2014). Гельминтофауна крупного рогатого скота в нахчыванской автономной республике. Международный Технико-Экономический Журнал, (2), 90-94.

[Farhadov, K.T.O. (2014). Helminth fauna of cattle in the Nakhchivan Autonomous Republic. Internation Technico-Economic Journal, 2, 90-94.]

Наджафов И.Г. (1989) научные основы усовершенствования тактики борьбы с тениаринхозом (на прим. АЗССР) автореферат дис. ... доктора медицинских наук / Ин-т мед. паразитологии и тропич. медицины им. Е. И. Марциновского. Москва, 1989

[Nadjafov, I G. (1989) Scientific basis for imporving the control of Taeniarhyncosis (in the Azerbaijan SSR). Summary of the dissertation for the Dr of Medical Sciences, E. I. Martsinov Institute of Parasitology and Tropical Medicine. Moscow 1989.]

Georgia (1 report)

Зиракишвили, Л. М., Иашвили, Н. Д., Иосава, М. Д., Поцхверия, Ш. О., & Месхи, М. В. (2009). Эпидемиологическая и эпизоотологическая характеристика трихинеллеза и тениаринхоза в Грузии. Теория и практика паразитарных болезней животных, 10, 172-176

[Zirakishvily L.M., Iashvily N.D., Iosava M.D., Pochveria Sh.O., Meschy M.V. Georgian S.S. (2009). Epidemiological and epizootological characteristics of Trichinella spp. and Taeniarhynchus saginatus infections in Georgia.Theory and Practice of Parasitic Diseases of Animals, 10, 172-176]

Iran (14 reports)

Abdi J , Karimi S. H. and Naserifar R. 2013. Zoonotic parasitic diseases in Ilam Province, Western Iran. African Journal of Microbiology Research. Vol. 7(23), pp. 2957-2960

Borji, H. And Parandeh, S. (2010). The abattoir condemnation of meat because of parasitic infection, and its economic importance: results of a retrospective study in north–eastern Iran. Annals of Tropical Medicine & Parasitology, 104, 641–647

Borji, H., Azizzadeh, M., and Kamelli, M. (2012). A retrospective study of abattoir condemnation due to parasitic infections: economic importance in Ahwaz, southwestern Iran. J. Parasitol., 98(5), 954–957

Eslami, A., Helan, J. A., & Gharouni, M. H. (2003). Report of two rare cases of rumen cysticercosis in cattle of Khorram‐Abad. Journal of Veterinary Research, 58(3), 267–270.

Faraji, R., Nazari, N., Negahdary, M. (2015). Prevalence of cysticercus of Taenia saginata in cattle slaughtered. Int J Res Med Sci. 2015 Jul;3(7):1662-1665

Garedaghi, Y et al. (2012) Prevalence of bovine cysticercosis of slaughtered cattle in Meshkinshahr Abattoir, Iran. Journal of Animal and Veterinary Advances, 11, 785-788.

Jahed Khaniki, G. R., Raei, M., Kia, E. B., Motevalli Haghi, A., Selseleh, M. (2010) Prevalence of bovine cysticercosis in slaughtered cattle in Iran. Tropical Anim health and Prod. 42, 141-143

Hamzavi, Y.,Nazari, N., Parandin, F., Ezzati, S., Faizi, F., Nourmohammadi, H., Bright A., Message, S. A. (2014). Harmful Importance and Determination of Cysticercosis Infection in Livestock Slaughtered at the slaughterhouse in Hamadan in 2013. Journal of Human and Animal Diseases 2 (2-4) 23-26. (In Persian)

Hashemnia, M., Shahbazi, Y., Afshari Safavi, E. A. (2015) Bovine Cysticercosis with Special Attention to Its Prevalence, Economic Losses and Food Safety Importance in Kermanshah, West of Iran. Journal of Food Quality and Hazards Control, 2, 26-29

Hosseinzadeh, S., Setayesh, A., Shekarforoush, S. S., Fariman, S. H. (2013) An epidemiological survey on the determination of Taenia saginata cysticercosis in Iran, using a PCR assay. Veterinary Record, 172, 451. doi: 10.1136/vr.101269

Mohammad Mirzaei, Ahmad Nematolahi, Javad Ashrafihelan, Hadi Rezaei. (2016)Prevalence of Infection with the Larval Form of the Cestode Parasite Taenia saginata in Cattle in Northwest Iran and its Zoonotic Importance. Turkiye Parazitol Derg. 40: 190-3

Nazari, N. (2016). Prevalence of Cysticercus bovis in cattle slaughter‐house in Piranshahr City, Iran (2011–2115). A research article presented at the International Congress on Biomedicine: Kermanshah University of Medical Sciences, Kermanshah, Iran.

Oryan, A., Goorgipour, S., Moazeni, M. and Shirian, S (2012) Abattoir prevalence, organ distribution, public health and economic importance of major metacestodes in sheep, goats and cattle in Fars, southern Iran. Tropical Biomedicine 29(3): 349–359

Oryan, A., Moghaddar, N., & Gaur, S. N. S. (1995). Taenia saginata cysticercosis in cattle with special reference to its prevalence, pathogenesis and economic implications in Fars Province of Iran. Veterinary Parasitology, 57(4), 319-327.

Kazakhstan (3 reports)

Кенжебаев С. А., Ибрагимов Д., Жумалиева Г. О. (2018) Эпизоотология (эпидемиология) гельминтозоонозов на юго-за-паде Республики Казахстан // Российский паразитологический журнал. 2018. Т. 43. № 2. С. 27–32. <https://doi.org/10.31016/1998-8435-2018-12-2-27-32>

[Kenzhebaev S. A, Ibragimov D., Zhumalieva G. O. (2018) Epizootology (epidemiology) of helminthozoonoses in the southwest of Republic of Kazakstan. Russian Journal of Parasitology. 2018; 12(2):27–32. [https://doi](https://doi/).org/10.31016/1998-8435-2018-12-2-27-32.]

Шонов Б.М. (1986) Особенности Эпизоотологии бовисного цистицеркоза крупного рогатого скота в Казахстане. Профилактика и меры борьбы с паразитарными болезями сельскохозяйственных животных в Казахстане. Сборник научных трудов. Восточнне отделение васхнил. Казахский научно-исследовательский ветеринарный институт. Алма-Ата ст. 94-100

[Shonov B.M. (1986) Features of Epizootology of bovis cysticercosis of cattle in Kazakhstan. Prevention and control measures against parasitic diseases of farm animals in Kazakhstan. Collection of scientific papers. East branch. Kazakh Veterinary Research Institute. Alma ata pp 94-100]

Шонов Б.М. (1985) Особенности Эпизоотологии бовисного цистицеркоза (финноза) крупного рогатого скота в Чимкентской области. Профилактика и меры борьбы с паразитарными болезями сельскохозяйственных животных в Казахстане. Сборник научных трудов. Восточнне отделение васхнил. Казахский научно-исследовательский ветеринарный институт. Алма-Ата ст. 126-130

[Shonov B.M. (1985) Features of Epizootology of bovis cysticercosis (finnoza) of cattle in the Chimkent region. Prevention and control measures against parasitic diseases of farm animals in Kazakhstan. Collection of scientific papers. East branch died. Kazakh Veterinary Research Institute. Alma-Ata pp 126-130]

Kyrgyzstan (1 report)

Шакиров, А. Б. (2004). Гельминты и гельминтозы крупного рогатого скота в Кыргызской Республике и меры борьбы с ними. Диссертация на соискание ученой степени доктора ветеринарных наук. Кыргызский Научно-Исследовательский Институт Животноводства, Ветеринарии И Пастбищ им. А. Дуйшеева. Бишкек, 282 ст.

[Shakirov, A. B. (2004). Helminths and helminth diseases of cattle in the Kyrgyz Republic and methods for their control. Dissertation for the Degree of Doctor of Veterinary Sciences. The A Dyishyeyeva Scientific Research Institute of Livestock, Veterinary and Pastures. Bishkek, 282 pp.]

Tadjikistan (2 reports)

Разиков, Ш. Ш., & Шерхонов, Т. (2011). Причины распространение бовисного цистицеркоза в зонах развитого яководства в Республики Таджикистан. Теория и практика паразитарных болезней животных, (12). 24-25

[Razikov, S. S. and Sherkhonov, T (2011) Causes of the distribution of bovine cysticercosis in the regions of animal breeding in the Republic of Tajikistan. Theory and Practice of Parasitic Diseases of animals, 12, 24-25.]

Разиков, Шомахмад Шерович (2011). Эпизоотологический анализ и меры борьбы с основными гельминто-зоонозами в Республике Таджикистан. Автореферат диссертации на соискание ученой степени доктора ветеринарных наук библиотеке гну вигис. Москва

[Razikov, S. S. (2011) Epizootiological analysis and method of control of major helmintjh zoonoses in the Republic of Tadjikistan. Dissertation, Doctor of Veterinary Sciences, GNU VIGIS Moscow. ]http://earthpapers.net/epizootologicheskiy-analiz-i-mery-borby-s-osnovnymi-gelminto-zoonozami-v-respublike-tadzhikistan#ixzz5eZUp2MEh

Turkmenistan

No articles found

Turkey (2 reports)

Fatma Selcan Kuş, Feride Kırcalı Sevimli, Özlem Miman (2013) Cysticercus bovis in Slaughtered Cattle in the Afyonkarahisar and Burdur Provinces and Its Importance from the Point of View of Public Health. Turkiye Parazitol Derg 2013; 37: 262-8

Fatma Selcan Kuş, Feride Kırcalı Sevimli, Özlem Miman (2014) Cysticercus bovis in Turkey and Its Importance from the Public Health Aspect. Turkiye Parazitol Derg, 38: 41-7

Uzbekistan (3 reports)

Dadaev, S. (no date) About the dfauna and biological clasification of cestodes of Uûngulates in Uzbekistan. http://guldu.uz/downloads/axborotnoma/pdf/2013-01-08.pdf

Гаипова, М. Э., Акрамова, Ф. Д., Сапаров, К. А., Азимов, Д. А., & Шакарбаев, У. А. (2016). Фауна и экология гельминтов крупного рогатого скота (Bos taurus dom.) Центрального Узбекистана. Российский паразитологический журнал, 4, 38.

[Gaipova, M.E., Akramova, F.D., Saparov, K.A., Azimov, D.A., & Shakarbayev, U.A. (2016). The fauna and ecology of cattle helminths (Bos taurus dom.) of Central Uzbekistan. Russian Journal of Parasitology, 4, 38.]

Ибрагимов ф.б. (1991) бовисный цистицеркоз и усовершенствование мер борьбы с ним автореферат дис. кандидата ветеринарных наук / самаркандский сельхоз. ин-т. Самарканд.

[Ibragimov, F. B. (1991) Bovines cysticercosis and improvemnet of control measures againt it. Dissertation for candidate of Veterinary Sciences. Samakand Agricultural Institute, Samarkand.]
